# Longitudinal Extensive Transverse Myelitis in an Immunocompetent Older Individual—A Rare Complication of Varicella-Zoster Virus Reactivation

**DOI:** 10.3390/medicina55050201

**Published:** 2019-05-23

**Authors:** Samar A. Abbas, Jeanine El Helou, Moussa A. Chalah, Hanine Hilal, Gaby Saliba, Halim Abboud, Samar S. Ayache

**Affiliations:** 1 Department of Neurology, Hôtel-Dieu de France Hospital, Faculty of Medicine, Saint-Joseph University, Beirut 1104-2020, Lebanon; samarabbas16@gmail.com (S.A.A.); jeaninelhelou@gmail.com (J.E.H.); halimabboud@yahoo.fr (H.A.); 2 EA 4391, Excitabilité Nerveuse et Thérapeutique, Université Paris-Est-Créteil, 94010 Créteil, France; moussachalah@gmail.com; 3 Service de Physiologie—Explorations Fonctionnelles, Hôpital Henri Mondor, Assistance Publique—Hôpitaux de Paris, 94010 Créteil, France; 4 Department of Neurology, Bellevue Medical Center University Hospital, Beirut 1104-2020, Lebanon; haninehilal@hotmail.com; 5 Department of Infectious Diseases, Hôtel-Dieu de France Hospital, Faculty of Medicine, Saint-Joseph University, Beirut 1104-2020, Lebanon; gebraelsaliba@gmail.com

**Keywords:** myelitis, varicella zoster virus, immunocompetence, transverse myelitis, longitudinal myelitis, herpes zoster

## Abstract

Varicella-zoster virus (VZV) is a human neurotropic herpes virus that causes chickenpox in children. After becoming latent in dorsal root ganglia, it can reactivate to cause dermatological manifestations, the most common one being shingles or herpes zoster. Severe neurologic dysfunctions can occur in immunocompromised patients such as encephalitis, meningitis, myelitis and neuropathy. Longitudinal extensive transverse myelitis (LETM) is an unusual neurological complication mainly described in immunocompromised patients, with very few cases described in immunocompetent ones. We hereby report a case of VZV-induced LETM in an immunocompetent older adult—a situation rarely described in the literature. LETM is a rare complication of VZV and its pathogenesis; therapeutic interventions and prognosis are far from being fully clarified. However, a prompt diagnosis is needed to allow a rapid initialization of treatment and ensure a better outcome. Although the therapeutic lines are not clear, immunosuppressive agents may have their place in cases of unsuccessful results and/or relapses following acyclovir coupled with a well conducted methylprednisolone therapy. Further studies are highly needed to improve the current understanding of the disease course and mechanisms, and to optimize therapeutic strategies.

## 1. Introduction

Varicella-zoster virus (VZV) is a human neurotropic herpes virus that causes chickenpox in children. After becoming latent in dorsal root ganglia, it can reactivate to cause herpes zoster or shingles that can potentially lead to post-herpetic neuralgia. Apart from this common presentation, reactivation of VZV can result in severe neurologic manifestations such as encephalitis, meningitis, and myelitis [1]. In the latter context, longitudinal extensive transverse myelitis (LETM) is an unusual severe neurological complication that has been mainly described in immunocompromised patients [1], with very few cases reported in immunocompetent ones [2,3,4]. Moreover, in a few cases, VZV-induced LETM was associated with anti-aquaporin-4 antibody serology suggesting a potential conversion to neuromyelitis optica spectrum (NMOS) disorder [5,6,7,8]. We hereby report a case of VZV-induced LETM in an immunocompetent individual—a situation rarely described in the literature.

Written informed consent was obtained from the patient to publish his data.

## 2. Case Presentation

A 77-year-old man, previously healthy, presented to the emergency department because of urinary retention, weakness and paresthesia of both lower limbs. The history went back to two weeks prior to presentation, where he started to complain of paresthesia and paresis of his right lower limb. The symptoms were of insidious onset and rapidly progressed to involve both lower limbs. The history was also marked by a zoster rash which appeared 10 days prior to the onset of neurological symptoms and involved the right L4–L5 dermatomes.

Upon admission, neurological exam revealed severe weakness of the lower limbs (medical research council (MRC) grade 0/5) and normal muscle strength of the upper limbs. Ankle and knee jerk reflexes were abolished bilaterally, and Babinski sign was found bilaterally. Furthermore, decreased sensation to touch was noticed with T2 sensitive level bilaterally. Vibration, temperature and pinprick sensations were also diminished in lower limbs. The remaining neurological functions were unremarkable.

**Figure 1:**
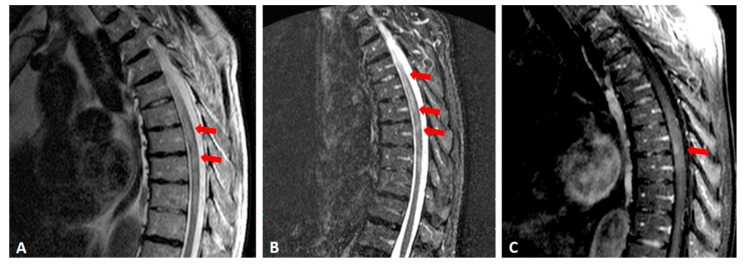
Magnetic resonance imaging showing a longitudinal hyperintense T2 lesion indicated by red arrows in the spinal cord extending from T2 to T11 (**A**,**B**) with gadolinium enhancement on T1 sequence at T7–T8 level (**C**).

Magnetic resonance imaging (MRI) revealed a hyperintense T2 lesion in the spinal cord extending from T2 to T11 (Figure 1A,B) with gadolinium enhancement on T1 sequence observed at the level of T7–T8 (Figure 1C). No brain or optic nerves lesions were found.

Laboratory tests revealed elevated white blood cells (WBC = 14100, 72% PMN) and positive IgG VZV serology. IgM VZV serology was negative. Other viral and bacterial serologies (Hepatitis B and C, HIV, CMV, HSV, Lyme disease and syphilis) were negative. Auto-immune and vitamins workup was unremarkable.

Cerebrospinal fluid (CSF) testing showed lymphocytic pleocytosis (94 cells/uL) and elevated VZV IgG (0.88) with a high VZV IgG index (14). The remaining tests (VDRL-TPHA test, Wright test, gram testing and bacterial cultures) were negative.

From these data, the diagnosis of VZV-induced LETM was made. Thus, the patient was treated with intravenous (IV) acyclovir (700 mg every 8 h (10 mg/kg)) for 21 days and methylprednisolone 1 g/day for three days followed by oral tapering. These treatments did not allow any clinical amelioration. Therefore, a five-day course of plasma exchange was performed and yielded unsatisfactory outcomes. An MRC grade of 1/5 muscle strength in lower limbs was achieved and sphincter dysfunction did not improve. The patient remained clinically stable without new manifestations for at least one year later (i.e., last follow-up).

## 3. Discussion

In this work, we report a case of acute LETM with extensive lesions of the thoracic spinal cord, caused by VZV reactivation. Our patient had a poor response to immunomodulatory therapy and was left with severe and disabling sequelae.

VZV is known to be responsible for a broad spectrum of neurological diseases [8]. The immune status of the patient influences the target, the spreading pathways and the clinical features of VZV-related complications [9,10]. While some such complications can be simple with benign prognoses (i.e., post-herpetic neuralgia), others are more challenging and can result in serious disability (i.e., myelitis, encephalitis).

Since its first description in 1876 [11], VZV-induced acute transverse myelitis (ATM) seems to occur in less than 1% of VZV reactivation situations [10] and usually affects immunocompromised patients [12,13]. Its occurrence in immunocompetent patients seems to be very rare. In this context, it is important to note that older age is associated with decreased cellular immunity which makes such patients only relatively immunocompetent [14]. Typical presentation of VZV-induced ATM consists of rapidly progressive motor, sensory and autonomic dysfunctions that are usually preceded by a dermatomal rash involving the same spinal level [15]. In an immunocompromised context, ATM can have an unusual MRI features or an atypical clinical presentation such as the absence of preceding shingles, or the presence of a myelopathy not limited to the level of zoster dermatome [10].

LETM is a rare variant of ATM and is defined by continuous lesions involving more than three spinal cord segments. It is characterized by acute catastrophic onset and poor outcome and was found to favor those with an immunocompromised background. Nevertheless, in rare cases, LETM–VZV has affected healthy immunocompetent subjects. The first occurrence was described in Japan in 2011, in an immunocompetent patient with P-ANCA glomerulonephritis, following a herpes zoster infection at the L2 territory, and was characterized by irregularly disseminated spinal lesions adopting a moth-eaten appearance of the cord on MRI [2]. A second case was depicted in Brazil, in a young healthy man, one week after a zoster rash, where the MRI features mimicked those of NMOS disorder [3]. Another particular case of hemorrhagic VZV LETM myelitis in a 15 year old Japanese adolescent was also reported [16]. Finally, a Japanese retrospective study has recently described the clinical and radiological characteristics of nine immunocompetent patients with VZV-related LETM [11].

The pathogenesis of LETM–VZV is unclear. Primary VZV infection stimulates the production of VZV antibody and activates VZV-specific T cells [10]. Afterwards, VZV remains latent in dorsal root ganglia and can reactivate later in life [10]. Myelitis is postulated to be the result of a direct viral invasion based on post-mortem data showing Cowdry type A intranuclear inclusions in spinal root ganglia and positive immunostaining for VZV antigens [10,16,17]. Besides the direct invasion hypothesis, vascular and allergic mechanisms were also proposed to intervene but to a lesser extent [10,16,17,18,19,20]. The potential post-infectious auto-immune reactions may include molecular mimicry between the infectious agent and central nervous system antigens as well as inflammatory processes secondary to microbial superantigens [18]. As for the vascular hypothesis, it was proposed by a few works that reported radiological features suggestive of vascular involvement [10,20].

Regarding the clinical examination, the typical presentation is a zoster lesion followed by myelopathy corresponding to the skin level [10]. Our patient did not have contiguity between the myelopathy and dermal levels. This finding is in line with the previous reports. A review of the available case reports on VZV myelitis has found that myelopathy level was distal from the zoster in five out of the 31 studied patients [10]. Some authors suggest that the virus at the origin of the zoster and the virus resulting in myelitis may arise at different spinal root ganglia [10]. An additional explanation for the discrepancy in myelitis and zoster levels is provided by the abovementioned vascular and immune hypotheses [10].

As for the laboratory testing, the diagnosis is usually based on serum (VZV IgG and IgM) and CSF (VZV IgG and/or VZV DNA) markers [21]. Our patient had high VZV IgG antibodies in his CSF. Although a VZV DNA test might have been helpful in this case, the high CSF IgG index confirmed the intrathecal secretion of the antibodies and could be sufficient by itself to set the diagnosis of VZV myelopathy [21].

Interestingly, some LETM–VZV reports suggest concomitant anti-aquaporin-4 antibodies with conversion to NMOS at a later follow-up. The possibility of such a conversion in our patient cannot be formally ruled out since serology tests were not done. However, this patient did not have any other brain or optic nerve lesions and remained stable with no further clinical symptoms in the last follow-up at one year. 

Regarding the management strategy, the optimal approach is still uncertain. The majority of cases were treated with antivirals (i.e., acyclovir) and steroids with variable outcomes. While unsatisfying improvement was found in some patients, partial or near full recovery was shown in others. The latter was reported in seven out of nine patients of the Japanese series. Interestingly, two of them relapsed and were then treated with chronic immunosuppressant therapy. Due to the limited improvement obtained with steroids, some teams have tried plasmapheresis, especially for those where an autoimmune mechanism has been incriminated in the development of this disease [11]. Again, response to plasma exchanges has shown great variability. In our patient, late pharmacological intervention might have been behind the poor clinical response. However, acyclovir-resistant VZV strains have been identified in immunocompromised individuals (such as in HIV-infected persons) and were found to be responsible for a very bad prognosis. Although our patient was relatively immunocompetent, the presence of such strains cannot be fully excluded [22].

## 4. Conclusions

To conclude, this case raises awareness of a rare complication of VZV in immunocompetent individuals. Although the pathogenesis and therapeutic interventions of LETM are far from being fully clarified, a prompt diagnosis is needed to allow rapid initialization of treatment and ensure a better outcome. In addition, despite the absence of clear therapeutic lines, recent data highlights the potential role of immunosuppressive agents in cases of unsuccessful results and/or relapses following acyclovir coupled with well conducted methylprednisolone therapy.

Finally, to improve the understanding and optimize the management strategies of such a challenging illness, the scientific and medical committees are obviously in need of additional research on this matter.
